# Synovitis, Acne, Pustulosis, Hyperostosis, and Osteitis (SAPHO): A Case Report

**DOI:** 10.7759/cureus.19636

**Published:** 2021-11-16

**Authors:** Amman Yousaf, Shoaib Muhammad, Basel Abdelazeem, Syed Intekhab Alam, Ahmed Mounir Elsyaed

**Affiliations:** 1 Internal Medicine, McLaren Flint, Flint, USA; 2 Radiology, Services Institute of Medical Sciences, Lahore, PAK; 3 Department of Urology, Ghulab Devi Hospital, Al-Aleem Medical College, Lahore, PAK; 4 Internal Medicine, McLaren Health Care, Flint, USA; 5 Musculoskeletal Radiology, Hamad Medical Corporation, Doha, QAT; 6 Orthopaedics, Weill Cornell School of Medicine - Qatar, Doha, QAT; 7 Orthopaedics Surgery, Hamad Medical Corporation, Doha, QAT

**Keywords:** synovitis, cellulitis, sapho, synovitis acne pustulosis hyperostosis osteitis, sapho syndrome

## Abstract

Synovitis, acne, pustulosis, hyperostosis, and osteitis (SAPHO) is an uncommon adulthood disorder that presents as focal swellings and pain accompanied by abnormal changes in bone and surrounding soft tissues. X-rays of the involved region are usually insignificant; however, CT and MRI show excellent visualization of the affected structures. Typical radiological images show cortical thickening leading to decreased marrow cavity, bony erosion, and ligament ossifications. Other associated findings are synovitis and joint effusions. It is usually diagnosed on the basis of clinical as well as radiological findings. The treatment initially relies on non-steroidal anti-inflammatory drugs (NSAIDs). Patients showing poor response are started on corticosteroids and disease-modifying antirheumatic drugs (DMARDs). We report two patients who presented with joint swellings. Their workup unmasked the underlying SAPHO, which was managed well with NSAIDs.

## Introduction

Synovitis, acne, pustulosis, hyperostosis, and osteitis (SAPHO) is a rare syndrome that presents in adults. It is comparable to chronic recurrent multifocal osteomyelitis (CRMO) in children. SAPHO was first reported by Chamot et al. in 1987, and only a few cases have been reported in the literature since then [1]. Typical presentations include local pain and swelling. Malignancy, infectious osteomyelitis, and arthritis are the top differentials. Due to its infrequent presentation, there is no established diagnosis criterion. Instead, clinical data and radiological information help make the diagnosis. Similarly, there are no management guidelines either. We present two cases of SAPHO along with their imaging data and discuss how they responded to various management options.

## Case presentation

Case 1 

**Figure 1:**
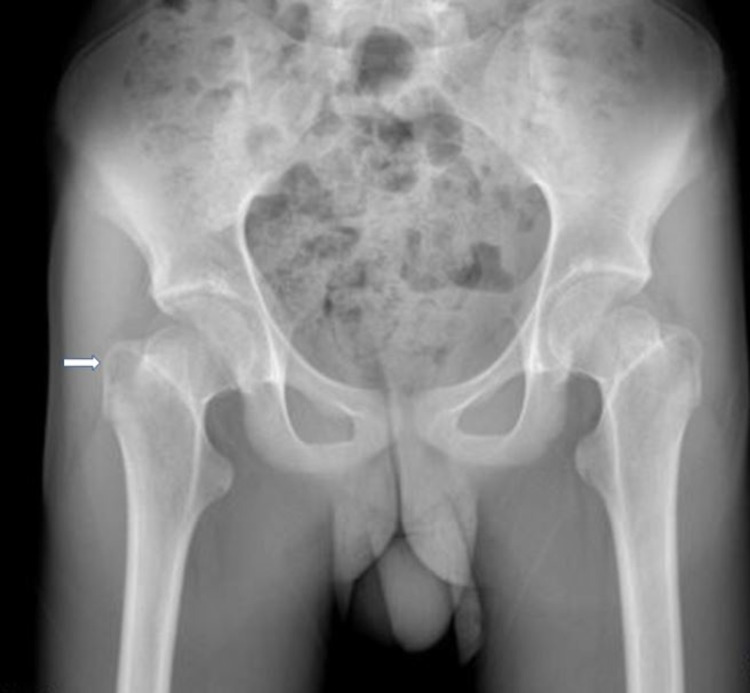
X-ray of the pelvis The image shows a subtle, non-specific, and well-defined lucency in the right trochanteric region (white arrow)

An 18-year-old male patient presented to the emergency department with right hip pain for two weeks. Examination revealed pain and mild to moderate tenderness in the right hip joint. The range of motion was decreased, and trying to initiate movement caused severe pain to the extent that the patient could not walk for gait assessment. Past history revealed similar but less severe episodes for the last four years. There was no history of fever, skin rash, or acne. His inflammatory laboratory investigations, including erythrocyte sedimentation rate (ESR) and C-reactive protein (CRP), were within the normal limits. X-rays of the pelvis and right thigh were unremarkable except for a small lucency in the right greater trochanteric region (Figure 1).

**Figure 2:**
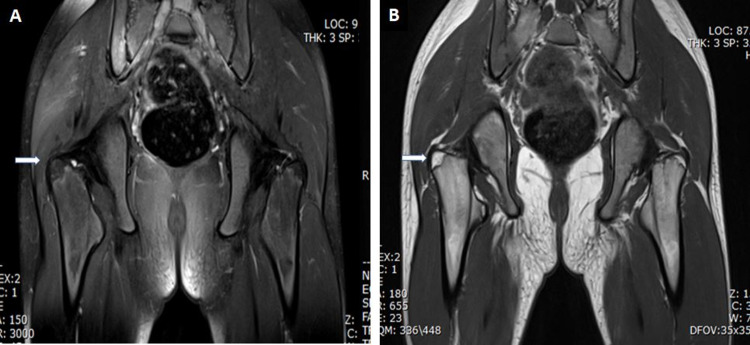
MRI pelvis with contrast A. Selected coronal proton density (PD) fat-sat image showing low signal intensity lesion involving the greater trochanter of the right femur (white arrow). B. Selected coronal T1-weighted image showing high signal intensity, well-defined lesion in the same region (white arrow) MRI: magnetic resonance imaging

Subsequently, an MRI of the right thigh showed trochanteric bursa effusion and right hip joint synovitis (Figures 2A, 2B).

Non-steroidal anti-inflammatory drugs (NSAIDs) and paracetamol were started for the management, and the patient showed excellent improvement for the first three months.

**Figure 3:**
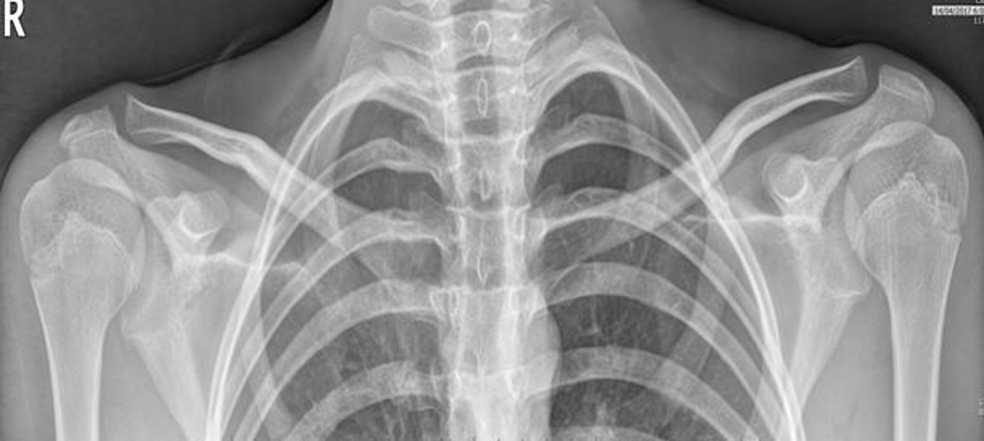
X-ray of the sternoclavicular joints The image displays the normal anatomical appearance of sternoclavicular joints and visualized osseous structures

He remained well for four months, after which he developed swelling of the right sternoclavicular joint. X-ray of the joint and laboratory investigations were found to be normal (Figure 3).

**Figure 4:**
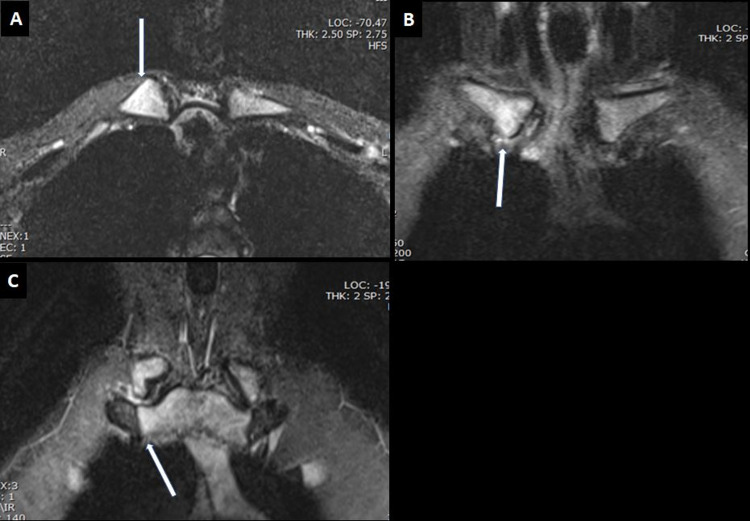
MRI of the sternoclavicular joints with contrast A. T1-weighted axial image showing abnormal marrow signal changes in the sternal end of the right clavicle (white arrow). B. T2-weighted coronal image demonstrating high abnormal signals in a similar area with mild right sternoclavicular effusion (white arrow). C. T1-weighted coronal image showing abnormal marrow signal changes in the right part of the manubrium sternum (white arrow) MRI: magnetic resonance imaging

Due to the previous history of the right hip joint, the lesion was investigated further with MRI (Figures 4A, 4B, 4C).

A patchy area of hyperintensity on T2 and hypointensity on T1 images close to the sternoclavicular junction was observed. On post-contrast images, there was a mild accentuated heterogeneous enhancement. Traces of fluid was also noted in the joint space, and marrow edema was seen along the articular margin and body of the sternum. MRI also showed subcutaneous edema and changes related to cellulitis in the overlying soft tissues. Clinical history and imaging data were suggestive of SAPHO syndrome. The patient was again started on paracetamol and NSAIDs based on the excellent previous response. At the one-month follow-up, the swelling was found to have improved. The patient was continued on the therapy and his symptoms resolved within three months.

Case 2

**Figure 5:**
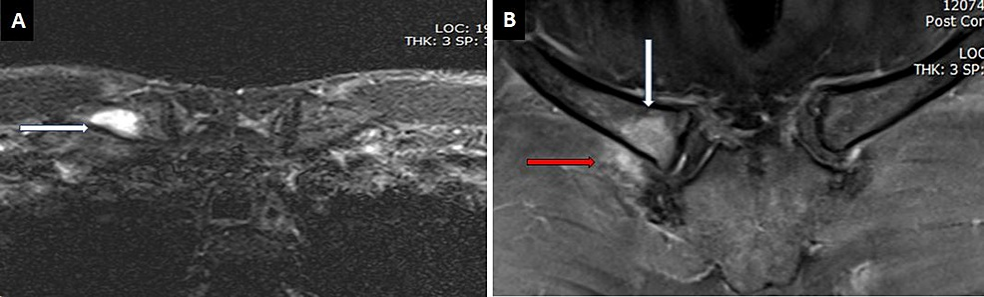
MRI of the sternoclavicular joint A. Selected axial T2-weighted image showing subchondral bone marrow edema (white arrow). B. T1-weighted gadolinium-enhanced image demonstrating enhancement in the medial end of the right clavicle (white arrow) with mild effusion of the right sternoclavicular joint, and surrounding soft tissue edema and enhancement (red arrow) MRI: magnetic resonance imaging

A 35-year-old male patient presented with a six-month history of pain in the anterior chest and neck. His pain initially had been mild but had become severe in the last three weeks. He was afebrile and did not have any constitutional symptoms. On examination, marked tenderness of the right sternoclavicular joint was noted. Initial laboratory investigations, tuberculosis workup, and chest X-rays were unremarkable. However, further study with MRI revealed subchondral bone marrow edema and enhancement involving the medial end of the right clavicle. In addition, mild effusion of the right sternoclavicular joint and surrounding soft-tissue edema was also seen (Figure 5A, 5B).

Given the patient's radiological and clinical findings, suspicion of SAPHO syndrome was raised. He was given an initial trial of paracetamol and NSAIDs and he showed remarkable improvement on his monthly follow-up visits. Though a biopsy was offered, the patient refused to undergo one and has shown no relapse to date thanks to the dramatic response to NSAIDs.

## Discussion

SAPHO syndrome is an autoinflammatory disorder in adults that closely resembles CRMO in children. It has been mainly reported in Japan and North Europe, with a predilection for the male gender [2]. Various inflammatory pathways have been hypothesized to play a role in the pathogenesis of this disease. Studies have demonstrated upregulated release of inflammatory cytokines coupled with increased neutrophil activation [3,4]. A recent study has stated that tissue necrosis factor-alpha (TNF-α), interleukin-8, interleukin-17, and interleukin-2B have a role in the development of SAPHO [5]. Another possibility is that in genetically predisposed subjects, *Propionibacterium acnes* infection can cause amplification of cytokines leading to SAPHO [6]. However, due to SAPHO being a rare entity, it has not been studied well.

Clinical symptoms are usually related to osteoarticular and dermatological manifestations. Significant complaints include local pain and swelling. Typically, osteoarticular changes appear first and are followed by skin changes. Around 25% of the patients lack any important dermatological manifestations, as was the case with our patients [7]. However, if present, females tend to have palmoplantar pustulosis (PPP), while males show severe acne. The osteoarticular changes usually seen are osteitis with sclerosis. Cortical thickening with hyperostosis and ligament ossification have also been reported. Periosteal and endosteal thickening coupled with cortical thickening may reduce the medullary cavity. Bony erosions similar to those seen in our patients can also be present [8]. The most frequent location is the anterior chest wall, as in both of our cases. Other significant sites include the sacroiliac joint, knees, ankles, and hip joint. One of our cases had a primary lesion in the right hip joint. SAPHO may mimic Paget's disease if it involves small joints.

The initial radiological investigation is typically an X-ray of the involved joint. However, radiographs usually show no significant findings. Therefore, a CT scan is favored to view bone-related changes. At the same time, an MRI can appreciate associated soft tissue changes like cellulitis and joint effusions. In our patient, MRI showed both of these changes. Technetium-99m scan can be utilized if CT and MRI are inconclusive. It can show a characteristic finding of the "bullhead sign" consistent with the upregulated activity of the sternoclavicular joint [9]. Analysis of the joint fluid is not recommended as it does not reveal any characteristic changes. Differential diagnoses include infectious osteomyelitis, malignancy, and arthritis. Due to overlapping symptoms, the diagnosis of SAPHO is difficult, particularly if skin changes are absent. Detailed clinical history and radiological findings are recommended to reach the diagnosis [10].

The treatment usually targets the presenting symptoms and helps prevent the disease from worsening. Typically, NSAIDs are considered first-line medications [11]. Both of our patients showed excellent improvement on NSAIDs. Disease showing resistance to NSAIDs is usually treated with corticosteroids, especially during flares. In cases where corticosteroids fail, management with disease-modifying antirheumatic drugs (DMARDs) is considered. Studies have shown them to be effective in many patients when given in the long term. Medications like TNF inhibitors are used if the disease is resistant to all the other usual drugs. Some studies have also demonstrated the use of anti-interleukin-1 and medicines related to interleukin-17 and interleukin-23. Yang et al. in 2018 reported the use of Janus Kinase (JAK) inhibitor to slow the progression of SAPHO, similar to rheumatoid and psoriatic arthritis [12]. The rationale behind this is that the upregulated culprit cytokines in SAPHO use the JAK receptor to exert their effects.

## Conclusions

SAPHO is a rare clinical syndrome that should be considered in adults presenting with multiple osteoarticular and skin changes including PPP, acne, osteitis, and sclerosis. Combined with clinical history, radiological modalities like CT scan and MRI provide excellent visualization and aid in making the diagnosis. Treatment modalities mostly include NSAIDs, corticosteroids, and DMARDs. We strongly recommend further studies to better understand the pathogenesis of the disease, which can promote further advancements in the treatment.
